# Factors associated with intrachoroidal cavitation and sinkhole formation in eyes with glaucomatous visual-field defects

**DOI:** 10.1007/s00417-023-06247-2

**Published:** 2023-10-04

**Authors:** Chiaki Yamaguchi, Naoki Kiyota, Naoki Takahashi, Yoko Takeda, Kazuko Omodaka, Satoru Tsuda, Toru Nakazawa

**Affiliations:** 1 Department of Ophthalmology, Tohoku University Graduate School of Medicine, 1-1 Seiryo-cho, Aoba-Ku, Sendai, Miyagi 980-8574 Japan; 2 Department of Ophthalmic Imaging and Information Analytics, Tohoku University Graduate School of Medicine, Sendai, Miyagi Japan; 3 Department of Retinal Disease Control, Tohoku University Graduate School of Medicine, Sendai, Miyagi Japan; 4 Department of Advanced Ophthalmic Medicine, Tohoku University Graduate School of Medicine, Sendai, Miyagi Japan

**Keywords:** Epidemiology, Glaucoma, Imaging, Intrachoroidal cavitation, Sinkhole

## Abstract

**Purpose:**

To investigate factors associated with intrachoroidal cavitation (ICC) and sinkhole formation in eyes with glaucomatous visual-field defects.

**Methods:**

This retrospective, cross-sectional study enrolled a total of 2808 eyes of 1482 patients who were diagnosed/treated for glaucoma and underwent swept-source optical coherence tomography (OCT). We first determined the prevalence of ICCs and sinkholes and their locations. Next, we selected one eye from each patient and compared the clinical characteristics of eyes with and without ICCs. Finally, in eyes with ICCs, we compared the clinical characteristics of eyes with and without sinkholes. Blood flow (BF), represented by laser speckle flowgraphy–measured tissue-area mean blur rate (MBR), was measured in the temporal optic nerve head (ONH), temporal peripapillary chorioretinal atrophy (PPA) zone, and in the ICC zone. ICC area and angle were analyzed in OCT en-face images. Mean deviation and total deviation in the central area (TD-central) were measured with Humphrey visual-field testing.

**Results:**

A total of 86 eyes (3.1%) had ICCs and 52 eyes (1.9%) had sinkholes. ICC eyes had a lower spherical equivalent and longer axial length (AL) than non-ICC eyes (*P* < 0.05). Patients with eyes with sinkholes were more elderly and had worse best-corrected visual acuity, worse TD-central, a larger ICC, and lower tissue-area MBR in the temporal ONH, temporal PPA zone, and ICC zone (*P* < 0.05).

**Conclusion:**

In eyes with glaucoma, AL elongation might be linked to ICC formation. Sinkhole formation might be associated with ICC enlargement, impaired ocular BF, and impaired retinal structure and function involving the central area.

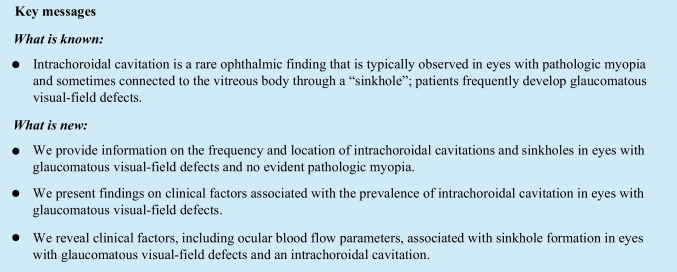

**Supplementary Information:**

The online version contains supplementary material available at 10.1007/s00417-023-06247-2.

## Introduction

Although it is rare, we sometimes observe intrachoroidal cavitations (ICCs) in eyes with myopia and/or glaucoma. An ICC is a yellow to orange crescent-shaped lesion typically located around the disc in funduscopic findings. Freund et al. [1] were the first to use optical coherence tomography (OCT) to study ICCs, and they reported that there was a crescent-shaped intrachoroidal void below the optic disc. At that time, they speculated that this finding was a peripapillary retinal pigment epithelium detachment. However, Toranzo et al. [2] used OCT with a higher resolution and found that this crescent-shaped lesion was an intrachoroidal hyporeflective space without retinal pigment epithelium detachment. Thus, they named this type of lesion “intrachoroidal cavitation.” The relationship between ICC formation and myopia is relatively well studied [3]. On the other hand, ICC formation in eyes treated for glaucoma has been poorly investigated, though it is known that ICC formation frequently leads to anatomically corresponding glaucomatous visual-field defects [4]. Glaucoma is the second leading cause of irreversible blindness worldwide and is associated with reduced quality of life [5, 6]. Although the only treatable risk factor for glaucoma is high intraocular pressure (IOP), many other risk factors have been suggested to be associated with glaucoma pathophysiology, including myopic stress to the optic nerve head (ONH) and impaired ocular blood flow (BF) [7–11]. Given this background, we hypothesized that there may be a link between ICCs, myopia, ocular BF, and glaucomatous visual-field defects.

Furthermore, it has been reported that ICCs sometimes develop a “sinkhole” formation, a condition in which peripapillary retinal tissue gradually sinks into a sclerochoroidal cavity associated with retinal hole formation and posterior vitreous prolapse [12, 13]. However, perhaps because sinkholes are a rare condition and need careful OCT observation to discover, their prevalence and location, as well as factors associated with their formation, are poorly studied.

Therefore, in this study, we first checked the etiological characteristics of ICCs and sinkholes in eyes that had glaucomatous visual-field defects. Next, we investigated risk factors, including axial length (AL) elongation, for ICC formation in eyes with glaucomatous visual-field defects. Finally, we investigated differences in clinical characteristics, including ocular BF parameters measured with laser speckle flowgraphy (LSFG), between ICC eyes with and without sinkholes.

## Subjects and methods

### Evaluation of ICCs and sinkholes using swept-source optical coherence tomography

To determine the prevalence of ICCs and sinkholes, we used 6 mm × 6 mm scans of the disc made with swept-source optical coherence tomography (SS-OCT; DRI OCT Triton, Topcon Co., Tokyo, Japan). In this study, ICCs were defined as an intrachoroidal hyporeflective space with a depth of 200 µm or more in the deepest B-scan image slice [14]. In each case, ICC depth was analyzed in the deepest slice showing an ICC. We also created optional en-face images, with the slice manually arranged to maximize ICC area and ICC angle at a desired layer and depth, using IMAGEnet6 software (ver 1.25.17169, Topcon) to better measure the ICC. Finally, we determined whether a sinkhole was present in the OCT B-scan images. Sinkholes were defined as a retinal hole formation with a posterior vitreous prolapse into the ICC, with reference to previous reports [12, 13]. The extent of the ICC and the sinkhole were recorded in two areas: one in clock-hour directions centered on the disc and one on a line connecting the center of the ONH and the fovea (i.e., the fovea-disc central axis). Circumpapillary retinal nerve fiber layer thickness (cpRNFLT) was obtained from the 6 mm × 6 mm OCT disc scans.

### Subjects

**Fig. 1:**
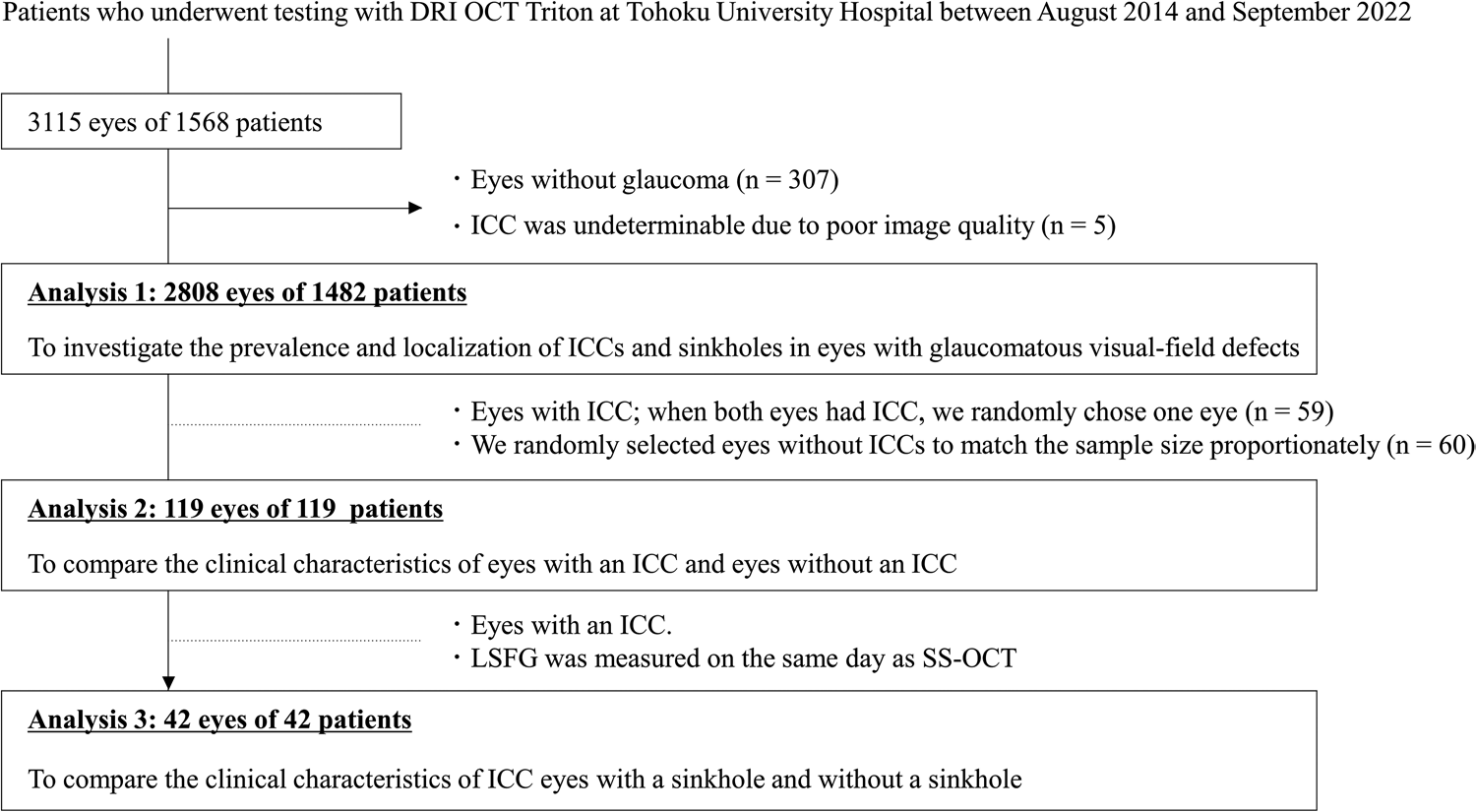
Selection scheme for eyes included in analyses 1 to 3. The dotted lines indicate the inclusion criteria and the horizontal arrows indicate the exclusion criteria

This retrospective, cross-sectional study reviewed the medical records of 3115 eyes of 1568 patients who underwent SS-OCT at Tohoku University Hospital, located in Miyagi, Japan, between October 2014 and September 2022. The initial diagnosis was made by a glaucoma specialist (TN) based on the presence of glaucomatous changes to the optic disc with corresponding visual-field defects matching the Anderson-Patella criteria [15]. Eyes were not diagnosed with glaucoma if they had characteristic findings of pathologic myopia, including diffuse chorioretinal atrophy, patchy chorioretinal atrophy, lacquer cracks, myopic choroidal neovascularization, and choroidal neovascularization-related macular atrophy [16]. After initial diagnosis, each patient underwent a medical history review, and baseline data were obtained for visual acuity and IOP with anti-glaucoma eye drops, as well as the results of slit lamp, gonioscopic, and dilated funduscopic examinations. A flow chart of the selection process for including eyes in the analysis is shown in Fig. 1. We excluded eyes without glaucomatous visual-field defects (*n* = 307) or if image quality was too poor to determine the presence of an ICC (*n* = 5). Through this process, we enrolled 2808 eyes of 1482 glaucoma patients. Diagnoses were as follows: normal-tension glaucoma (NTG): 49.4% (1387 eyes); primary open-angle glaucoma (POAG): 30.6% (858 eyes); and other glaucoma types (primary angle-closure glaucoma or secondary glaucoma): 20.0% (563 eyes). We carefully checked these patients’ SS-OCT disc scan data to identify the prevalence or localization of ICCs and sinkholes, as described above. Next, among 2808 eyes of 1482 glaucoma patients, we identified 86 eyes with ICCs of 59 patients. If both eyes had an ICC, we generated random values for both eyes and selected the eye with the higher random value, resulting in a sample of 59 eyes with ICCs from 59 patients and 1482 eyes without ICCs from 1482 patients. To obtain a proportionate sample size for group comparisons, we assigned random values to these 1482 eyes without ICCs and selected the 60 eyes with the highest values. Thus, we compared the clinical characteristics of eyes with an ICC (*n* = 59) and eyes without an ICC (*n* = 60). Finally, we compared the clinical characteristics, including BF parameters, of eyes with an ICC and a sinkhole (*n* = 28) and eyes with an ICC without a sinkhole (*n* = 14) that underwent LSFG measurement on the same date as the SS-OCT scan. Since the majority of glaucoma cases in Japan are NTG [17] and the study was conducted at a university hospital, there may have been a bias towards the inclusion of patients that were severely affected by non-IOP factors.

### Measurement of clinical variables

IOP was measured with Goldmann applanation tonometry, AL was measured with the IOL Master (Zeiss Meditec, Dublin, CA, USA), and the visual field was measured with the SITA standard 24-2 program of the Humphrey Field Analyzer (HFA) (Carl Zeiss Meditec, Dublin, CA, USA); only reliable measurements of the visual field were used (fixation errors < 20%, false positives < 33%, and false negatives < 33%). Total deviation (TD)-central in this study indicates the averaged value for TD in the central field of the Garway-Heath sector map [18].

Ocular BF was assessed with the LSFG-NAVI device (Softcare Co., Ltd., Fukutsu, Japan), which measures mean blur rate (MBR) in arbitrary units (AU). First, an ellipsoid band was manually drawn around the ONH to define the region of interest (ROI) in composite MBR color maps. The accompanying LSFG software (LSFG Analyzer, version 3.1.59.0) then automatically divided the large-vessel and tissue (i.e., capillary) areas of the ROI and determined vessel-area MBR and tissue-area MBR separately [19]. Tissue-area MBR has been reported to be a good indicator of BF in the deep ONH [20, 21]. We set another ROI, which was slightly larger than the ROI for the ONH and was superimposed over it and aligned on the nasal edge, and then used the LSFG analysis software to subtract the larger ROI from the ROI for the ONH, thereby obtaining a measurement of tissue-area MBR in the crescent PPA zone [10]. Additionally, in this study, we set a novel ROI around the ICC area and subtracted the ROIs of other areas from it, thereby obtaining a measurement of tissue-area MBR in the ICC zone. If ICCs were found in multiple sites, the largest ICC was used for BF assessment. Before the LSFG measurements, the patient’s pupils were dilated with 0.4% tropicamide, a muscarinic antagonist (Mydrin M; Santen Pharmaceutical Co., Ltd). After the instillation, the patients sat in a dark, quiet room for 15 min to stabilize pupil dilation, blood pressure (BP), and pulse rate (PR). Then, BP and PR were measured (HBP-1300; Omron) and LSFG was performed.

### Statistical analysis

All data are shown as the mean ± standard deviation. When values for a clinical parameter were missing on the date that SS-OCT was performed, the value measured at the closest date to the SS-OCT examination was used for the analysis. For group comparisons, the Shapiro-Wilk test was used to test for normality. For continuous variables following a normal distribution, the t-test was employed. For variables not adhering to a normal distribution, we used the Wilcoxon rank sum test. For nominal variables, Pearson’s chi-squared test was used. An analysis of covariance (ANCOVA) was used as a sensitivity analysis to adjust for age to determine statistical differences in the background clinical factors of ICC eyes with and without a sinkhole. The receiver operating characteristic curve was used to determine the power of AL to discriminate between eyes with and without an ICC. The correlations between BF parameters were analyzed with Spearman’s rank correlation coefficient. All statistical analyses were performed with R software version 4.1.3 (available at https://www.R-project.org/).

## Results

In 2808 eyes with glaucomatous visual-field defects, the prevalence of ICCs was 3.1% (86 eyes) and sinkholes were observed in 52 eyes (1.9% in total and 60.5% of ICC eyes). Four eyes had two sinkholes, and one eye had three sinkholes. As shown in Supplementary Figure S1, both ICCs and sinkholes were most frequently observed in the 6 o’clock direction, while 21.4% of ICCs were present at the fovea-disc central axis. This ratio was approximately 4 times higher in the ICC eyes with sinkholes than in the ICC eyes without sinkholes (28.5% vs. 7.1%). A histogram of AL of the eyes with ICCs is provided in Supplementary Figure S2. Among the eyes with ICCs, 25 eyes (29.1%) were not highly myopic (AL < 26.5 mm).

Among 119 eyes of 119 patients, the characteristics of the eyes with ICCs compared to the eyes without ICCs were as follows: NTG was more common (83.1% vs. 40.0%, *P* < 0.001), spherical equivalent (SE) was lower (−7.42 ± 3.24 vs. −4.50 ± 4.43, *P* < 0.001), and AL was longer (27.41 ± 1.46 vs. 25.49 ± 1.88 mm, *P* < 0.001) (Table 1). The cut-off value for AL to discriminate eyes with ICCs from eyes without ICCs was 26.59 mm (AUC = 0.80).

**Table 1:** Differences in clinical characteristics of glaucoma eyes with and without an ICC

Variables	ICC (-)	ICC ( +)	*P* value
Number of patients	60	59	−
Glaucoma type (NTG:POAG:others)	24:12:24	49:9:1	< 0.001^†,*^
Age, years	57.71 ± 14.80	58.30 ± 14.01	0.850
Male:female	27:33	27:32	> 0.999^†^
Systolic BP, mmHg	131.94 ± 19.67	132.08 ± 19.38	0.972
Diastolic BP, mmHg	77.30 ± 12.11	77.52 ± 12.44	0.927
Pulse rate, bpm	72.75 ± 12.18	74.61 ± 11.70	0.589^‡^
Hypertension (n %)	20 (33.3%)	13 (22.0%)	0.241^†^
Diabetes mellitus (n %)	5 (8.3%)	4 (6.8%)	> 0.999^†^
Dyslipidemia (n %)	15 (25.0%)	14 (23.7%)	> 0.999^†^
Heart disease (n %)	10 (16.6%)	6 (10.2%)	0.441^†^
BCVA, logMAR	0.04 ± 0.29	0.09 ± 0.32	0.171^‡^
Spherical equivalent, diopters	−4.50 ± 4.43	−7.42 ± 3.24	< 0.001^‡,*^
IOP, mmHg	14.77 ± 3.47	14.31 ± 3.41	0.376^‡^
CCT, μm	525.77 ± 54.78	522.29 ± 41.10	0.998^‡^
Axial length, mm	25.49 ± 1.88	27.41 ± 1.46	< 0.001^‡,*^
CpRNFLT, μm	66.17 ± 21.40	72.14 ± 14.73	0.079
MD, dB	−10.26 ± 8.81	−7.44 ± 6.59	0.135^‡^

*BP* blood pressure, *CCT* central corneal thickness, *cpRNFLT* circumpapillary retinal nerve fiber layer thickness, *IOP* intraocular pressure, *MD* mean deviation, *NTG* normal tension glaucoma, *POAG *primary open angle glaucoma

Unmarked *P* value: t test. † Chi-square test. ‡ Wilcoxon rank sum test. * indicates statistical significance

**Fig. 2:**
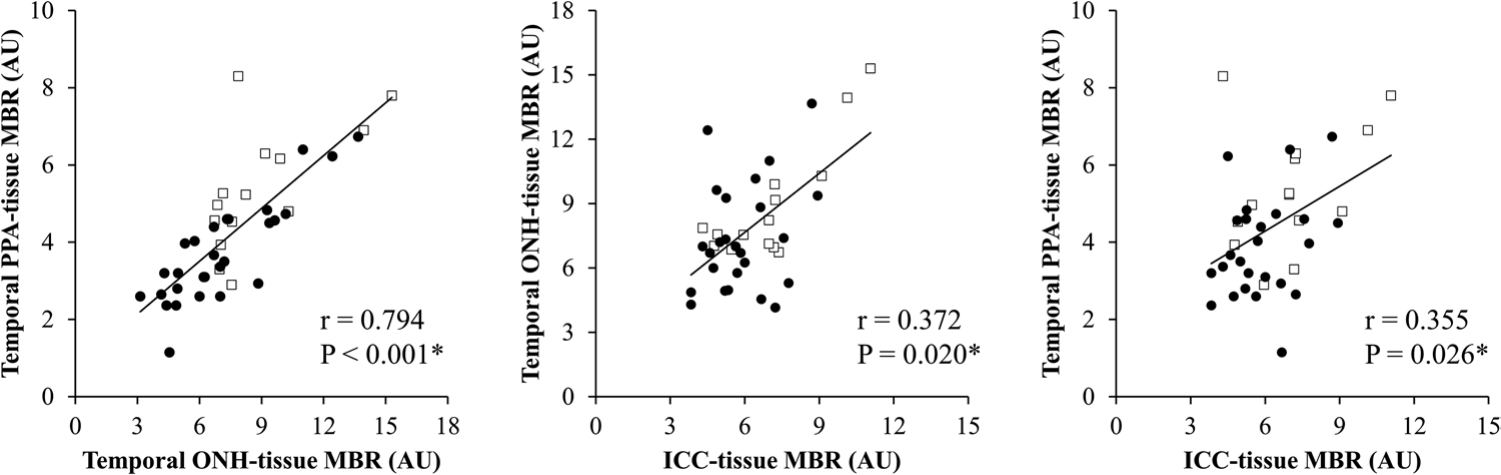
Scatterplots showing the relationship between temporal ONH-tissue MBR, temporal PPA-tissue MBR, and ICC-tissue MBR. Correlations were determined with Spearman’s correlation coefficient. Asterisks indicate statistical significance. There were significant correlations between temporal PPA-tissue MBR and temporal ONH-tissue MBR (r = 0.794, *P* < 0.001), temporal ONH-tissue MBR and ICC-tissue MBR (r = 0.372, *P* = 0.020), and temporal PPA-tissue MBR and ICC-tissue MBR (r = 0.355, *P* = 0.026). The black-rimmed white squares indicate data from ICC eyes without a sinkhole, and the black circles indicate data from ICC eyes with a sinkhole

**Fig. 3:**
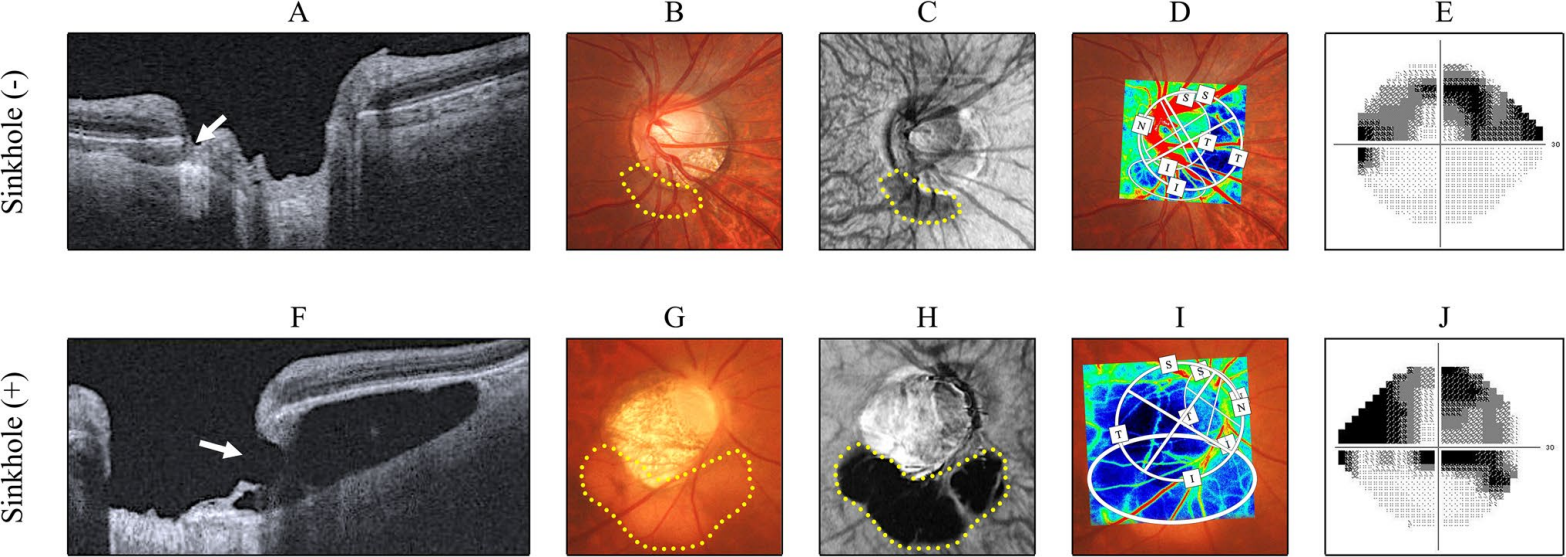
From left to right: representative optical coherence tomography (OCT) B-scan images, fundus photographs, OCT en-face images, laser speckle flowgraphy color map images overlayed on the corresponding fundus photos, and grayscale images of the visual field (Humphrey Field Analyzer; 24-2 Swedish interactive threshold algorithm). **A-E** The upper panels show data from a 22-year-old male glaucoma patient with an ICC and no sinkhole. BF in the ONH and the ICC zone was relatively good, and the central visual field was spared. Data for this patient were as follows: axial length, 27.74 mm; IOP, 12 mmHg; BP, 106/62 mmHg; pulse rate, 70 bpm; MD, -11.40 dB; TD-central, -6.83 dB; temporal ONH-tissue MBR, 9.90 AU; temporal PPA-tissue MBR, 6.16 AU; ICC-tissue MBR, 7.20 AU; ICC area, 1.76 mm^2^; ICC depth, 0.27 mm; and ICC angle, 68.5 degrees. **F-J** The lower panels show data from a 50-year-old male glaucoma patient with an ICC and a sinkhole. BF in the ONH and the ICC zone was impaired, and a central visual field defect was present. Data for this patient were as follows: axial length, 25.89 mm; IOP, 14 mmHg; BP, 122/84 mmHg; pulse rate, 85 bpm; MD, -2.64 dB; TD-central, -13.74 dB; temporal ONH-tissue MBR, 5.63 AU; temporal PPA-tissue MBR, 3.31 AU; ICC-tissue MBR, 6.00 AU; ICC area, 5.72 mm^2^; ICC depth, 0.45 mm; and ICC angle, 112.9 degrees. The yellow dots indicate the ICC zone in the fundus photographs and OCT en-face images. The white arrows indicate the sinkhole in the B-scan OCT images

In 42 eyes with ICCs, sinkholes were observed in 28 eyes (66.7%). As shown in Table 2, The characteristics of the eyes with ICCs and sinkholes versus the eyes with ICCs and no sinkholes were as follows: older age (62.89 ± 10.77 vs. 50.70 ± 12.45 years, *P* = 0.003), worse best-corrected visual acuity (BCVA; 0.20 ± 0.40 vs. −0.09 ± 0.05 logMAR, *P* < 0.001), lower cpRNFLT (68.61 ± 13.61 vs. 84.38 ± 10.36 μm, *P* < 0.001), worse MD and TD-central (−9.11 ± 6.81 vs. -3.38 ± 2.45 dB, *P* = 0.003 and −10.93 ± 9.25 vs. −3.17 ± 4.57 dB, *P* = 0.003, respectively), lower temporal ONH-tissue MBR (7.09 ± 2.61 vs. 8.90 ± 2.68 AU, *P* = 0.018), lower temporal PPA-tissue MBR (3.74 ± 1.31 vs. 5.35 ± 1.58 AU, *P* = 0.002), larger ICC area (3.66 ± 2.54 vs. 1.81 ± 0.65 mm^2^, *P* = 0.002), a deeper ICC (0.41 ± 0.13 vs. 0.33 ± 0.12 mm, *P* = 0.021), and a wider ICC angle (95.95 ± 42.05 vs. 70.00 ± 26.65 degrees, *P* = 0.048). We confirmed that these differences were statistically significant after age adjustment (*P* < 0.05). The difference in ICC-tissue MBR between the groups neared significance before age adjustment (*P* = 0.072) and reached significance after age adjustment (*P* = 0.039). Neither AL nor SE differed significantly between the groups (27.52 ± 1.29 vs. 27.25 ± 1.70 mm, *P* = 0.679 and −7.27 ± 3.62 vs. −8.03 ± 2.41 diopters, *P* = 0.739, respectively). Figure 2 shows that BF parameters were correlated with each other in the eyes with ICCs (r = 0.355–0.794, *P* < 0.05). Figure 3 shows a representative ICC eye without a sinkhole, and an ICC eye with a sinkhole.

**Table 2:** Differences in clinical characteristics of ICC eyes with and without a sinkhole

Variables	Groups		*P* value	Age-adjusted *P* value
	Sinkhole (-)	Sinkhole ( +)		
Number of eyes	14	28		
Glaucoma type (NTG:POAG:others)	12:2:0	23:4:1	> 0.999^†^	
Age, years	50.70 ± 12.45	62.89 ± 10.77	0.003^*^	
Male:female, n	5:9	15:13	0.444^†^	
Systolic BP, mmHg	132.69 ± 23.22	133.58 ± 18.94	0.777	
Diastolic BP, mmHg	75.23 ± 13.55	79.62 ± 13.52	0.276	
Pulse rate, bpm	73.08 ± 14.12	73.76 ± 8.63	0.459	
Hypertension, *n*	3 (21.4%)	8 (28.5%)	0.723^†^	
Diabetes mellitus, *n*	1 (7.1%)	2 (7.1%)	> 0.999^†^	
Dyslipidemia, *n*	5 (35.7%)	6 (21.4%)	0.459^†^	
Heart disease, *n*	1 (7.1%)	3 (10.7%)	> 0.999^†^	
BCVA, logMAR	−0.09 ± 0.05	0.20 ± 0.40	< 0.001^*^	0.009^*^
IOP, mmHg	13.94 ± 2.53	14.75 ± 4.10	0.688^‡^	
Spherical equivalent, diopters	− 8.03 ± 2.41	− 7.27 ± 3.62	0.739^‡^	
CCT, μm	509.50 ± 23.02	526.14 ± 43.97	0.191^‡^	
Axial length, mm	27.25 ± 1.70	27.52 ± 1.29	0.679	
CpRNFLT, μm	84.38 ± 10.36	68.61 ± 13.61	< 0.001^*^	< 0.001^*^
MD, dB	− 3.38 ± 2.45	− 9.11 ± 6.81	0.003^‡*^	0.005^*^
TD-central, dB	− 3.17 ± 4.57	− 10.93 ± 9.25	0.003^‡*^	0.006^*^
ONH-tissue MBR, AU	11.65 ± 2.70	9.26 ± 3.06	0.025^*^	0.018^*^
Temporal ONH-tissue MBR, AU	8.90 ± 2.68	7.09 ± 2.61	0.018^*^	0.045^*^
PPA-tissue MBR, AU	6.35 ± 1.49	4.29 ± 1.56	< 0.001^*^	0.007^*^
Temporal PPA-tissue MBR, AU	5.35 ± 1.58	3.74 ± 1.31	0.002^*^	0.001^*^
ICC-tissue MBR, AU	7.04 ± 1.98	5.87 ± 1.41	0.072	0.039^*^
ICC area, mm^2^	1.81 ± 0.65	3.66 ± 2.54	0.002^‡,*^	0.011^*^
ICC depth, mm	0.33 ± 0.12	0.41 ± 0.13	0.021^‡,*^	0.048^*^
ICC angle, degree	70.00 ± 26.65	95.95 ± 42.05	0.048^‡,*^	0.043^*^

*AU* arbitrary units, *BCVA* best-corrected visual acuity, *BP* blood pressure, *CCT* central corneal thickness, *cpRNFLT* circumpapillary retinal nerve fiber layer thickness, *ICC* intrachoroidal cavitation, *IOP* intraocular pressure, *MD* mean deviation, *MBR* mean blur rate, *NTG* normal tension glaucoma, *POAG *primary open angle glaucoma, *TD* total deviation

Unmarked *P* value: t test or ANCOVA. † Chi-square test. ‡ Wilcoxon rank sum test. * indicates statistical significance

## Discussion

This retrospective study included 2808 eyes of 1482 patients who underwent SS-OCT and had visual-field defects that were classified as glaucomatous. We examined each SS-OCT image from these eyes and found that the prevalence of ICCs was approximately 3.0%. Among eyes with an ICC, a sinkhole was found in approximately 60% of cases. We randomly selected 119 eyes of the 119 patients and found that the eyes with ICCs had a higher rate of NTG diagnosis, lower SE, and longer AL. The patients with ICCs and a sinkhole were older than the patients with ICCs without a sinkhole. After age adjustment, the eyes with ICCs had worse TD-central and BCVA, a larger ICC area, deeper ICCs, a wider ICC angle, and lower tissue-area MBR in the temporal ONH, temporal PPA zone, and ICC zone.

The prevalence of ICCs in this study was relatively low, although it is within the range of previous reports (2.2–12.8% [22–25]). This low prevalence might only be apparent in comparison with previous reports, a majority of which focused on highly myopic patients [2, 3, 14]. However, Yeh et al. [26] reported that ICCs are not exclusively observed in eyes with pathologic myopia. Our results support this, given that 25 of 86 eyes with ICCs had low myopia (< 26.5 mm). Therefore, although the prevalence was not high compared to eyes with pathologic myopia, attention must still be paid to ICC formation in eyes with glaucomatous visual-field defects but no evident characteristic findings of pathologic myopia. One potential explanation for ICC development without high myopia is that myopic mechanical stress does not uniformly affect the posterior eye. If the AL of the eye focally extends towards the ONH, the mechanical stress on the ONH can be intense, but this effect may not be fully reflected in AL measurements, which are usually made from the central cornea to the foveal axis. As a result, the impact of myopia could be underestimated in some cases. A 3D evaluation of the posterior eye might be useful to clarify this point in the future [27].

The finding that ICCs were typically observed inferior to the ONH is consistent with previous reports [4, 28]. In addition, sinkholes were also typically observed on the inferior edge of the ONH. We speculate that AL elongation towards the posterior pole typically puts tensile stress on the inferior edge of the ONH, leading to disruption of Jacoby’s line and choroidal dropout in this area (i.e., ICC development), in addition to myopic torsion and tilting of the ONH and temporal shift of Bruch’s membrane (i.e., γ-PPA development) [29]. Presumably, the structure of the edge of the ONH in the 6 o’clock direction might be more susceptible to these biomechanical forces, and the retinal nerve fibers, as well as Jacoby’s line, are more likely to be disrupted (i.e., sinkhole formation).

In this study, ICCs were observed more often in NTG eyes than eyes with POAG or other types of glaucoma. Other than in NTG, it is likely that elevated IOP is the main cause of the development of glaucomatous visual-field defects. On the other hand, IOP is within the normal range in NTG eyes, so glaucoma pathogenesis is perhaps also related to IOP-independent factors, such as myopic mechanical stress (including ICC development) in the ONH. Thus, our results might be understandable.

Sinkhole formation was observed in approximately 60% of the ICC eyes and was associated with older age, a larger ICC area, a deeper ICC, and a wider ICC angle. In contrast, sinkhole formation was not associated with AL elongation. Previously, only Wei et al. [3] have counted the number of sinkholes in ICC eyes (they were found in 13 of 16 ICC eyes); to our knowledge, no report has shown risk factors for sinkhole formation. Based on our results, we speculate that even if AL elongation stops, chronic mechanical stress over time and tissue fragility due to aging could lead to RNFL disruption and the prolapse of vitreous fluid into the ICC (i.e., sinkhole formation). After a sinkhole has formed, vitreous fluid enters the choroidal cavitation via the sinkhole and may further enlarge the ICC in area, depth, and angle.

Interestingly, our results also show that BCVA, the central visual-field, and ocular BF parameters were impaired in ICC eyes with a sinkhole. Some glaucoma patients show parafoveal scotomas even at an early stage, and suggested risk factors for this include myopia and BF-associated factors [30–33]. We previously examined this so-called “vascular theory” and found that temporal ONH-tissue MBR and temporal PPA-tissue MBR are correlated with each other; reduction of these parameters contributes to BCVA loss and central visual-field defect progression in eyes with glaucoma and a myopic disc [9, 10, 34]. This study suggests that BF impairment associated with ICCs and sinkholes might also affect BF in the adjacent ONH. Using OCT angiography, Kim et al. [35] reported that microvascular dropout was entirely proximally adjacent to the peripapillary ICC. We speculate that chronic mechanical stress caused by myopia might develop into an ICC that is then exacerbated by sinkhole formation, which extends their areas and reduces BF. This results in hypoperfusion in the ONH, including in the temporal quadrant, impairing axons passing through this region and leading to structural and functional deterioration in the retina, including in the central area. Alternatively, impaired BF around the ONH and sinkhole formation may just be secondary changes to mechanical stress around the ONH. The causal relationship between BF and neurodegeneration, ICC, and sinkhole formation can only be clarified by collecting time-series data of these events.

Our study has significant clinical implications. First, our results show that ICCs with sinkholes tend to lead to central visual-field defects and impaired visual acuity, indicating a poor disease prognosis. When we treat eyes with glaucomatous visual-field defects, it is important to look not only for signs such as cupping or nerve fiber layer thinning in OCT scans, but also to carefully check for the presence of ICCs and sinkholes. We may consider intensifying anti-glaucoma treatment at an earlier stage than usual if we treat the eye for glaucoma. Second, our study offers a starting point for discussing whether it is appropriate to treat eyes with glaucomatous visual-field defects accompanied by an ICC as having glaucoma, or whether we need to establish a different disease concept. Our findings suggest that it may be possible to organize a novel disease concept around keywords like myopic mechanical stress and/or ocular blood flow impairment. Further investigation is needed to clarify this point in the future.

This study has several limitations: the first and most important is the retrospective, cross-sectional, single-center design. Thus, the cause and consequence relationship between the presence of an ICC or sinkhole and associated factors cannot be determined. It was also unclear when the ICCs and sinkholes formed in our patients and whether the ICCs were currently expanding. These factors cannot be determined without large-scale OCT data from health examinations. Second, since LSFG is a two-dimensional imaging technique and there are no studies that have set the ROI around the ICC, it is unclear to what extent ICC-tissue MBR reflects BF in the ICC zone. The third limitation is that we did not perform multivariable regression analyses of many explanatory variables; we only adjusted for age, which differed significantly between the groups, in the analysis shown in Table 2. This was due to the small sample size, as ICCs and sinkholes are rare findings; we also had to consider multicollinearity caused by correlations between ocular parameters. Therefore, we have avoided discussing which differences in parameters were more important based on our results, and instead propose that the differences in characteristics between the two groups that we observed are initial findings. We believe that these findings may justify a future study with a more robust statistical model and a larger sample size. The fourth limitation of this study is that, although we focused on patients who were diagnosed with and treated for glaucoma, it is challenging to determine whether these cases were truly “pure” glaucoma or whether they should have been considered as ICC eyes with glaucomatous visual-field defects. Our goal was to distinguish these two conditions, but to do so, we first need to investigate to what extent ICCs can be observed in patients diagnosed with and treated for glaucoma in real-world data and what differences exist in their clinical backgrounds. We believe that research needs to continue in this direction, and that there needs to be a deeper discussion regarding this disease concept in the future. The fifth limitation is the small sample size following the selection process illustrated in Fig. 1. Initially, we had 1,482 non-ICC eyes, but for a proportionate group comparison, we randomly chose 60 non-ICC eyes. When we set α = 0.05, the effect sizes detectable at a power of 0.8 in our sample size (i.e., 59 ICC eyes vs. 60 non-ICC eyes) were 0.53 and 0.52 for the Wilcoxon rank sum test and t-test, respectively. Therefore, it is essential to note that, given this sample size, it is not possible to argue for the absence of differences in parameters with smaller effect sizes, such as cpRNFLT or MD (0.32 and 0.36, respectively). Given that ICCs are rare and challenging to detect, while it might be difficult at present, further research involving larger samples is needed. Nonetheless, the difference in AL demonstrated in this analysis had a very high effect size of 1.13. As such, we believe our sample size was sufficient for detecting AL differences.

In conclusion, although they are rare, ICCs and sinkholes can be found in eyes diagnosed with and treated for glaucoma. We found that ICC formation was associated with AL elongation in these eyes. Moreover, if a sinkhole develops, the ICC might enlarge, leading to a severe visual-field defect involving the central area accompanied by BF impairment around the ONH. Therefore, ICC and sinkhole formation might be involved in the pathophysiology of a specific glaucoma subgroup or might be an independent disease concept.

### Supplementary Information

ESM 1 (PNG 2422 kb)

High Resolution Image (TIF 10663 kb)

ESM 2 (PNG 113 kb)

High Resolution Image (TIF 1092 kb)
