# Supplementary figures and images for: Factors associated with intrachoroidal cavitation and sinkhole formation in eyes with glaucomatous visual-field defects
Source: Graefes Arch Clin Exp Ophthalmol. 2023 Oct 4;262(2):557–66. doi: 10.1007/s00417-023-06247-2 (PMC10844383; doi:10.1007/s00417-023-06247-2)

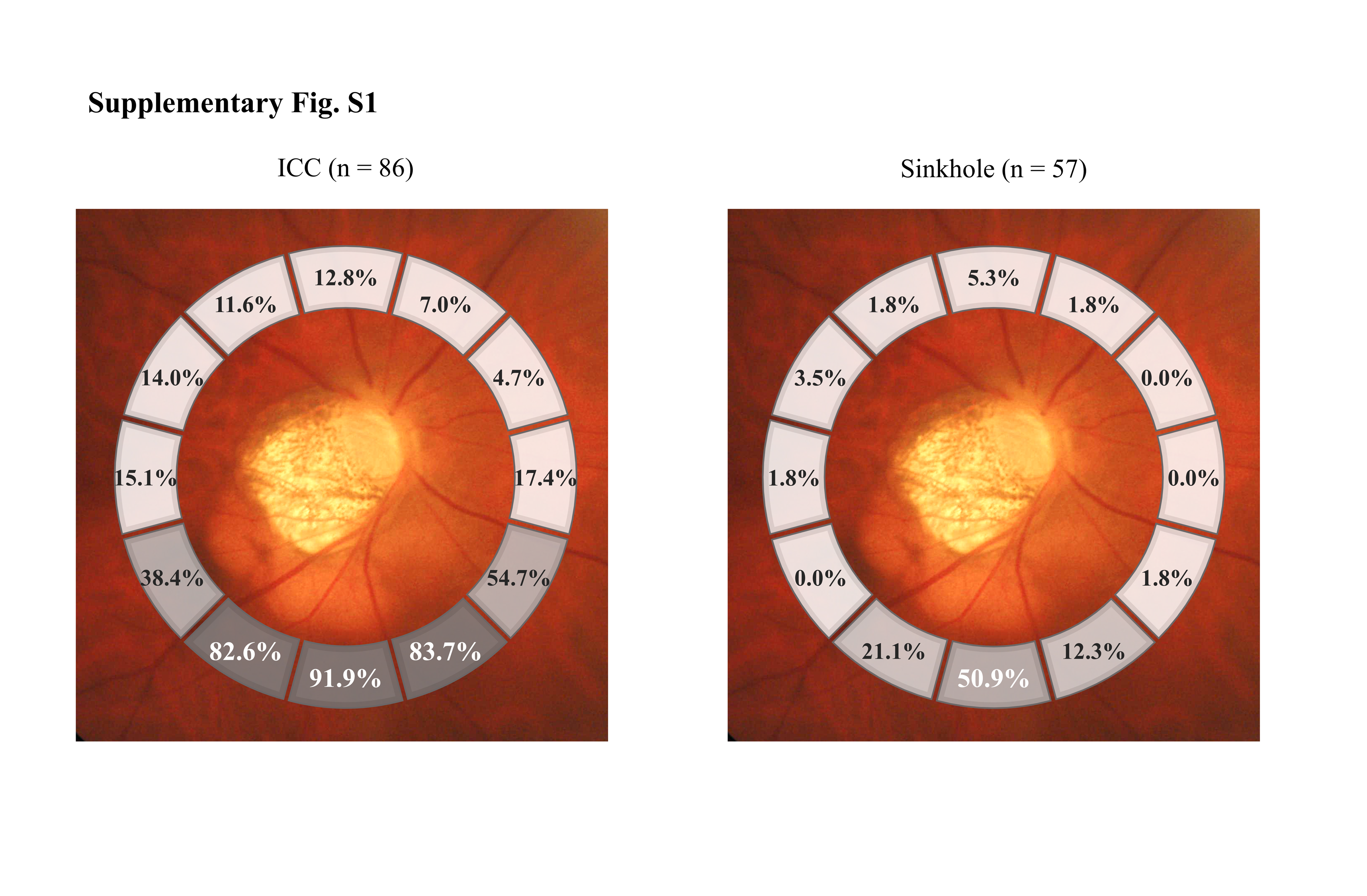

Supplement: Supplementary file 1 — (PNG 2422 kb) [file 417_2023_6247_Fig4_ESM.png]

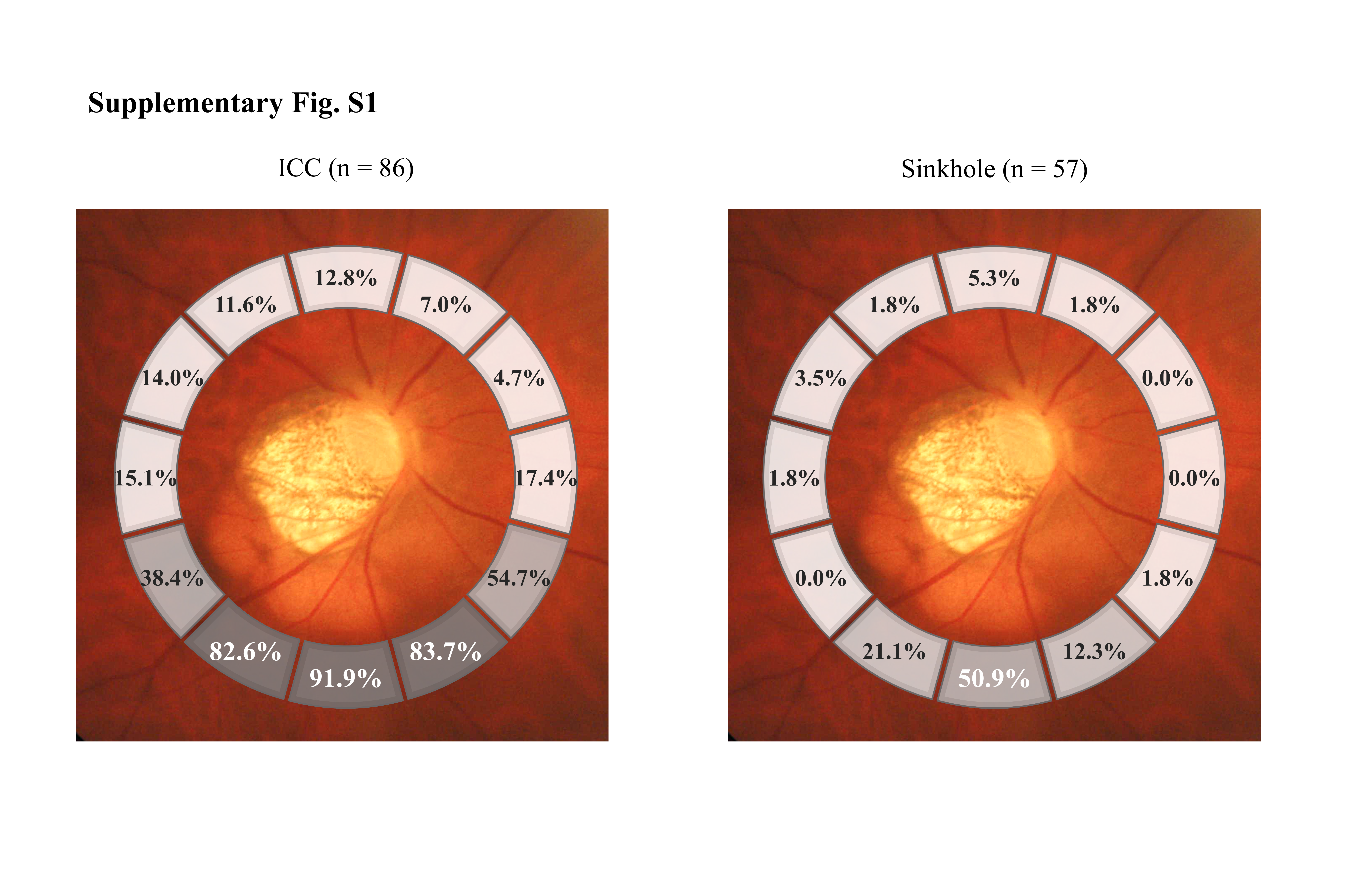

Supplement: Supplementary file 2 — High Resolution Image (TIF 10663 kb) [file 417_2023_6247_MOESM1_ESM.tif]

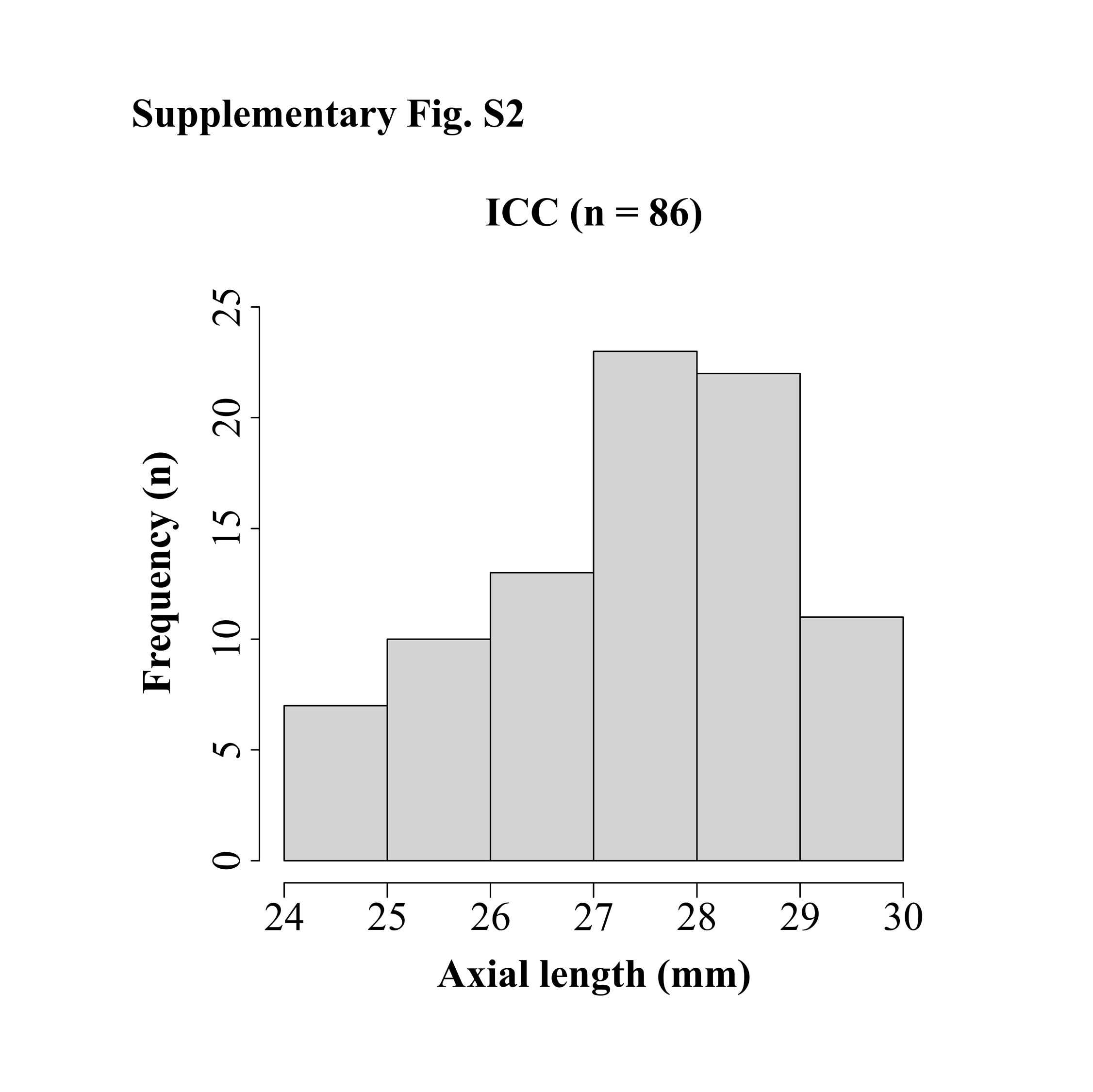

Supplement: Supplementary file 3 — (PNG 113 kb) [file 417_2023_6247_Fig5_ESM.png]

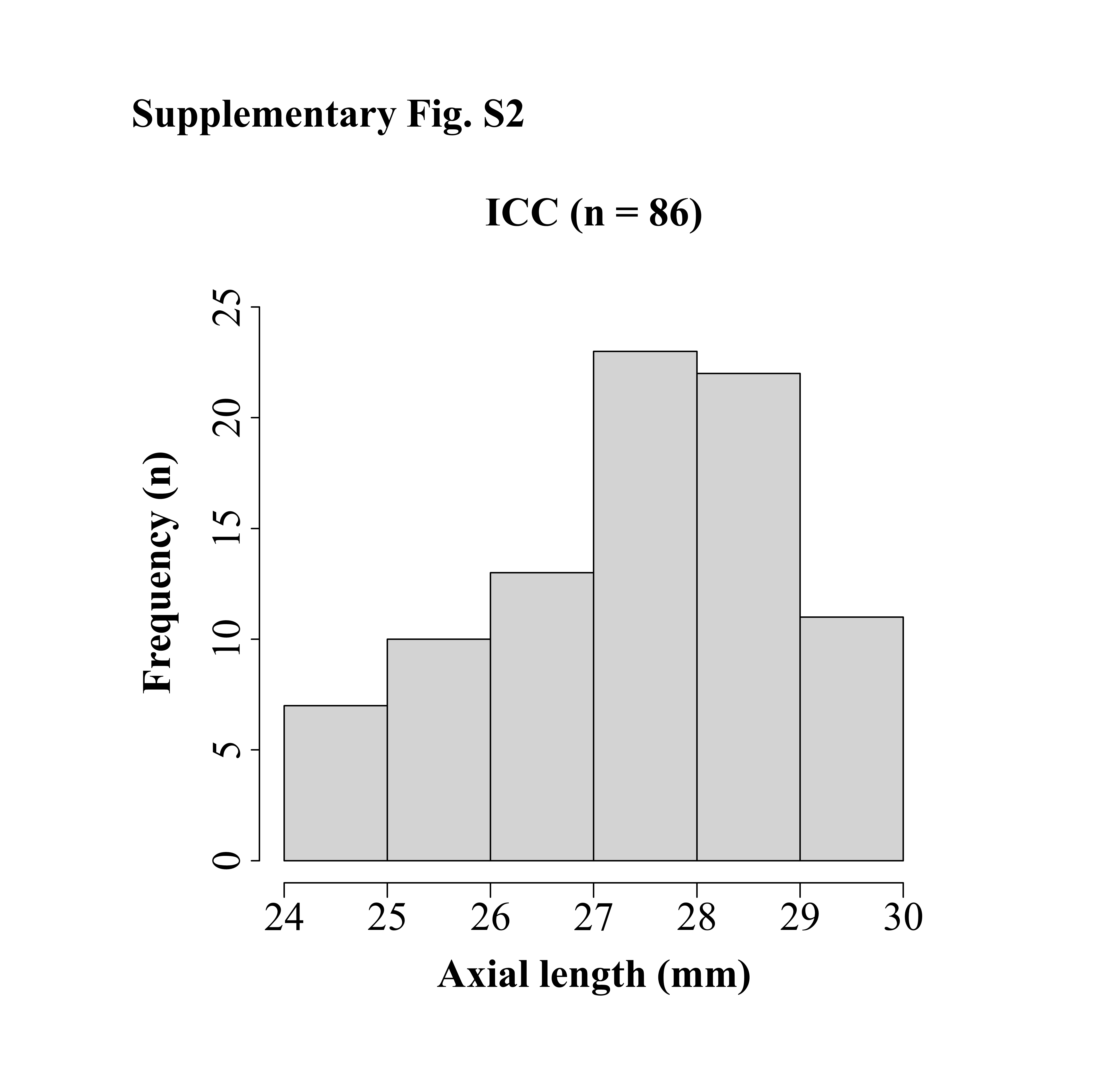

Supplement: Supplementary file 4 — High Resolution Image (TIF 1092 kb) [file 417_2023_6247_MOESM2_ESM.tif]
